# Characteristics of individuals not visiting their primary care provider

**DOI:** 10.1186/2045-4015-3-40

**Published:** 2014-11-28

**Authors:** Asaf Bitton, Sagar B Dugani

**Affiliations:** Division of General Medicine and Primary Care, Brigham and Women’s Hospital, 1620 Tremont St, Rm 3-002P, Boston, Massachusetts 02120 USA; Department of Health Care Policy and Center for Primary Care, Harvard Medical School, Boston, USA; Ariadne Labs, a Joint Center for Health Systems Innovation, Brigham and Women’s Hospital and Harvard School of Public Health, Boston, USA; Department of Medicine, Brigham and Women’s Hospital, 75 Francis Street, Boston, Massachusetts 02115 USA

## Abstract

The observational study by Rosen and colleagues described the proportion and characteristics of individuals who do not visit their primary care physician regularly. Overall, they identify a very low rate of non-attendance, high rates of visit frequency, and describe predictors of non-attendance. In this study of 421,012 individuals, only 6,217 (or, 1.5% of the study population) did not visit their primary care physician over the four-year study period. Multivariate analysis showed that the strongest predictors of non-attendance were being male, being a new immigrant, and the presence of fewer chronic diseases. This study raises important questions about why patients seem to be so engaged with primary care in Israel, whether this engagement explains part of the Israeli health system’s success, and ways to best structure primary care services in the future.

Primary care providers [PCP] play a central role in the provision of comprehensive, preventive, and coordinated healthcare. There is significant debate on the relationships between the frequency, contribution, and cost-effectiveness of the different types of visit modalities and the effective provision of primary care. Understanding the magnitude and correlates of patient non-attendance to PCP visits is important when weighing the best options for structuring scarce primary care resources.

In their study, Rosen and colleagues [1] conducted a retrospective observational study to determine the proportion and characteristics of individuals who do not regularly visit their PCP in Israel. The authors reviewed the records of 421,012 members of one district of Clalit Health Services, the largest health maintenance organization in Israel, and documented the number of visits to their PCP over the four-year study period. Notably, they found that only 6,217 [1.5% of study population] did not visit their PCP during the four-year study period. Multivariate analysis revealed that the strongest predictors of non-attendance were being male, having fewer chronic diseases, and being a new immigrant to Israel.ss

Overall, this article addresses three important points. First, it describes the proportion and characteristics of those who do not regularly see their PCP. Second, it identifies a group of individuals who may potentially benefit from outreach efforts –new immigrants– who are less likely to see their PCP. Finally, it provides a foundation for characterizing the consequences of long-term non-attendance to the PCP, both for patients and health policy makers seeking to transform primary care.

In this study, 98.5% of individuals had seen a PCP at least once in four years, which is higher than the 94.9% seen in an earlier study among the elderly in Israel [2], 91% of Canadians who have a regular source of care [3], and 91.3% seen among general practice in England [4]. Notably, Israeli patients in the study by Rosen and colleagues had very high average numbers of annual primary care visits (7 to 8) suggesting a frequency of nearly 1 visit every 7 weeks, quite high for patients with an average of under 1 chronic disease.

How do we explain this apparent high utilization of primary care services and low rates of non-attendance? A few possibilities emerge. It could indicate high levels of patient engagement with a functioning primary care system that is clearly defined as the the first contact point for non-emergent entry in the Israeli health care system. Importantly, each Clalit member is clearly assigned to one PCP, who also serves as a gatekeeper to most other specialties. This clear *empanelment* of patients to a defined provider and practice ensures that patients have a usual source of care and access point. These factors could result in higher continuity and high PCP attendance rate in this population. Notably, measures of continuity of primary care in Israel (67%-81%) [5] are higher than those seen in the United States (50%-60%) [6] and other high-income countries.

The low rate of PCP non-attendance in the present study might be interpreted somewhat cautiously, as the study members were generally young with low levels of chronic diseases, factors that might have been associated with fewer PCP visits. Further, in the present study it is unclear if non-attenders alternately obtained care at clinics outside of their health maintenance organization, or if they had higher resultant use of emergency room services [7], though this is unlikely in the Israeli health care system. In addition, it is unclear whether certain minority groups outside of immigrants to Israel (such as Israeli Arabs) were adequately represented in this sample. Strong evidence exists that Israeli Arabs have significantly lower rates of preventive gynecologic care [8], lower quality diabetes care [9], and lower rates of cancer screening tests compared to the Israeli Jewish population [10].

If in fact the findings of the study by Rosen and colleagues can be generalized to the broader Israeli primary care system, a number of further questions arise. Are the high number of PCP visits and broad engagement of the vast majority of the population to primary care an important, and potentially under-recognized factor in Israel’s ability to provide a higher value health system (higher quality at lower costs) than many of its high-income country counterparts such as the US and Switzerland? Here it would be helpful to compare total expenditures and human resource allocation to primary care between these countries, as a broad literature suggests that countries with broader spending on, and orientation toward, primary care achieve better outcomes at lower costs [11]. Additionally, factors that may explain Israeli patients’ high use of the primary care system may include positive patient experiences, the habituation to primary care physicians as the focal entry point of the health system, and limited use of nurse triage to keep patients from contacting their physicians. Other financial and structural features promoting primary care use include the absence of co-payments for primary care visits and insurance models that require primary care physician preauthorization for an extensive array of procedures and referrals. Further granular policy and historical understanding of the organizational, political, and financial structures that have allowed Israel to preferentially direct spending and human resources toward primary care (instead of hospitals) would be useful to policymakers and researchers in other high income countries.

Finally, even with this high level of support and utilization within primary care, Israeli policymakers and planners must ask whether inevitably scarce resources are being deployed in the most effective manner? Using physicians to see patients on average every 7–8 weeks is unlikely to be consistent with “top of license” physician work (i.e. seeing the most complex patients and managing teams of providers who can see less acute patients). As the patient centered medical home movement in the United States is beginning to show, restructuring the primary care workforce into physician-managed teams of providers that better utilize information technology, asynchronous visits (for example, email, telephone), and proactive chronic disease registries has the potential to improve primary care outcomes [12]. The United States can clearly learn from the strategies in Israel that have successfully engaged patients with primary care services as the first point of usual and continuous care, thereby lowering costs and driving higher levels of quality. In turn, Israel may do well to learn and adapt new models of team-based care that spread the work across a more diffuse array of providers, enabling physicians to drive forward complex care management, oversee registry-based chronic disease management, and engage more frequently in quality improvement efforts.

## Authors’ information

Asaf Bitton, MD MPH is based at the Division of General Medicine and Primary Care, Brigham and Women’s Hospital, and Ariadne Labs.

Sagar B. Dugani, MD PhD is based at Brigham and Women’s Hospital, and Ariadne Labs.

## Commentary on

Rosen et al. Low rate of non-attenders to primary care providers in Israel- a retrospective longitudinal study. *Israel Journal of Health Policy Research* 2014 **3**:15.
